# Effects of Randomized Controlled Infancy-Onset Dietary Intervention on Leukocyte Telomere Length—The Special Turku Coronary Risk Factor Intervention Project (STRIP)

**DOI:** 10.3390/nu13020318

**Published:** 2021-01-22

**Authors:** Niina Pitkänen, Katja Pahkala, Suvi P. Rovio, Outi J. Saijonmaa, Anna E. Nyman, Antti Jula, Hanna Lagström, Jorma S. A. Viikari, Tapani Rönnemaa, Harri Niinikoski, Olli Simell, Frej Fyhrquist, Olli T. Raitakari

**Affiliations:** 1 Research Centre of Applied and Preventive Cardiovascular Medicine, University of Turku, 20520 Turku, Finland; niina.pitkanen@utu.fi (N.P.); suvrov@utu.fi (S.P.R.); tuula.simell@gmail.com (O.S.); olli.raitakari@utu.fi (O.T.R.); 2 Centre for Population Health Research, University of Turku and Turku University Hospital, 20520 Turku, Finland; hanlag@utu.fi (H.L.); hjniin@utu.fi (H.N.); 3 Auria Biobank, University of Turku and Turku University Hospital, 20520 Turku, Finland; 4 Paavo Nurmi Centre, Sports & Exercise Medicine Unit, Department of Physical Activity and Health, University of Turku, 20520 Turku, Finland; 5 Minerva Institute for Medical Research, 00290 Helsinki, Finland; outi.saijonmaa@helsinki.fi (O.J.S.); nymanannae@gmail.com (A.E.N.); frej.fyhrquist@gmail.com (F.F.); 6 Department of Public Health Solutions, Institute for Health and Welfare, 20750 Turku, Finland; antti.jula@fimnet.fi; 7 Department of Public Health, University of Turku and Turku University Hospital, 20520 Turku, Finland; 8 Department of Medicine, University of Turku, 20520 Turku, Finland; jorvii@utu.fi (J.S.A.V.); erkron@utu.fi (T.R.); 9 Division of Medicine, Turku University Hospital, 20520 Turku, Finland; 10 Department of Physiology and Department of Pediatrics, University of Turku, 20520 Turku, Finland; 11 Department of Clinical Physiology and Nuclear Medicine, Turku University Hospital, University of Turku, 20520 Turku, Finland

**Keywords:** telomere length, telomere attrition, dietary intervention, childhood, genetics, risk factors

## Abstract

Reduced telomere length (TL) is a biological marker of aging. A high inter-individual variation in TL exists already in childhood, which is partly explained by genetics, but also by lifestyle factors. We examined the influence of a 20-year dietary/lifestyle intervention on TL attrition from childhood to early adulthood. The study comprised participants of the longitudinal randomized Special Turku Coronary Risk Factor Intervention Project (STRIP) conducted between 1990 and 2011. Healthy 7-month-old children were randomized to the intervention group (*n* = 540) receiving dietary counseling mainly focused on dietary fat quality and to the control group (*n* = 522). Leukocyte TL was measured using the Southern blot method from whole blood samples collected twice: at a mean age of 7.5 and 19.8 years (*n* = 232; intervention *n* = 108, control *n* = 124). Yearly TL attrition rate was calculated. The participants of the intervention group had slower yearly TL attrition rate compared to the controls (intervention: mean = −7.5 bp/year, SD = 24.4 vs. control: mean = −15.0 bp/year, SD = 30.3; age, sex and baseline TL adjusted β = 0.007, SE = 0.004, *p* = 0.040). The result became stronger after additional adjustments for dietary fat quality and fiber intake, serum lipid and insulin concentrations, systolic blood pressure, physical activity and smoking (β = 0.013, SE = 0.005, *p* = 0.009). A long-term intervention focused mainly on dietary fat quality may affect the yearly TL attrition rate in healthy children/adolescents.

## 1. Introduction

Telomeres are structures consisting of DNA sequence repeats and associated proteins that protect the ends of eukaryotic chromosomes. Telomere length (TL) decreases progressively in most proliferating somatic cell types as a result of terminal DNA sequence loss associated with replication of linear chromosomes [1]. In stem cells, TL is maintained by the activity of telomerase, a ribonucleoprotein enzyme, which adds sequence repeats to the ends of chromosomes [1]. Once telomeres become critically short, cell enters replicative senescence and/or apoptosis [1].

An inverse relationship between age and TL exists, and on average telomeres are longer in women than in men [2]. However, substantial inter-individual variation exists and can be observed from birth/infancy [3,4,5]. Other predictors of the inter-individual variation in TL include paternal age, genetic factors and ethnicity [2,5,6,7,8]. Additionally, several life-style related factors, such as body mass index (BMI), smoking, physical activity and diet, have been suggested to influence TL. In general, adherence to healthy lifestyle associates with longer leukocyte TL [9], while shorter TL is associated with many aging-related complex diseases, such as cardiovascular diseases and type 2 diabetes [10,11,12]. One possible mechanism behind the link between lifestyle related risk factors and TL shortening may be inflammation and oxidative stress, that are associated with poor lifestyle and have, in addition to DNA replication, been related to accelerated shortening of telomeres [13,14].

Several cross-sectional and longitudinal studies have suggested that dietary factors and patterns are related to TL, but the results are inconclusive [15,16,17,18,19,20,21]. Some studies have highlighted the role of overall healthy dietary patterns [9,20], while others have shown more specific effects for nutrients, including fiber and fatty acids, and for foods, comprising processed meat and deep fried potato product intake [15,16,17,18,22]. Particularly in childhood, the effects of diet and lifestyle on TL are largely unknown. The globally unique infancy-onset Special Turku Coronary Risk Factor Intervention Project (STRIP) study provides an exceptional possibility to tackle this lack of knowledge [23]. An intense dietary counseling of a heart-healthy diet has been given for 20 years for the intervention group, and concomitantly, the study provides a vast data bank on diet and other factors related to cardiometabolic health. The STRIP study has demonstrated that a long-term and repeated intervention targeting particularly on replacement of saturated fat with unsaturated fat in the diet has resulted in a plethora of phenotypic changes in the intervention children—all effects pointing to a reduced risk of atherosclerotic diseases and diabetes [24,25,26]. We therefore hypothesized that the low saturated fat oriented dietary/lifestyle intervention could associate with leukocyte TL from childhood to early adulthood.

## 2. Materials and Methods

### 2.1. The Special Turku Coronary Risk Factor Intervention Project Cohort

The STRIP study is a prospective randomized controlled dietary and lifestyle intervention trial that started in 1990. Healthy infants and their families living in Turku, Finland were recruited by nurses at well-baby clinics during a routine 5-month visit between December 1989 and May 1992. Of the 1880 eligible families, altogether 1062 participated. The infants were randomized into an intervention (*n* = 540) and a control (*n* = 522) group at age 7 months by random numbers (group allocation unmasked). The recruitment protocol, study design and data collection methods have been previously described in detail [23]. Attrition analyses between the study participants and those lost to follow-up have been repeatedly conducted showing no differences in key characteristics, such as weight, total cholesterol, blood pressure (BP) or saturated fat intake [23,27,28]. The study was approved by the Joint Commission on Ethics of Turku University and Turku University Central Hospital. Written informed consent was obtained from the parents at the beginning of the study and from the children when they were 15 and 18 years old.

### 2.2. Intervention and Assessment of Diet, Smoking and Physical Activity

The families in the intervention group received individually tailored dietary counseling at 1–3-month intervals until the child was 2 years old and biannually thereafter. The families in the control group were met biannually until age 7 years and annually thereafter. The intervention was based on the latest Nordic Nutrition Recommendations available at the given time with main focus on the replacement of saturated fat with unsaturated fat [29]. The targeted monounsaturated and polyunsaturated to saturated fatty acid ratio (P + M)/S was 2:1 and a cholesterol intake < 200 mg/day. Additionally, the counseling targeted reduction of salt intake and encouraged the use of fruit, vegetables and whole-grain products. The counseling was given to the parents until the child was 7 years old and after that, it was increasingly directed to the child. The nutritionist-led counseling sessions were individualized in that the sessions were based on the child’s diet as assessed through food records, and they also addressed specific dietary questions of the families. A fixed diet was never ordered. Each counseling session had a specific dietary topic and involved performing tasks; the intervention children were, for example, taught to identify high saturated fat foods and shown how to replace these with foods with a more favorable fat composition. The intervention phase continued until the participants were 20 years old. The control group received only basic health education. Similar measurements were performed in both groups.

Food consumption was assessed using 3-to-4-day food records (including one weekend day) that were checked for accuracy by a nutritionist. The participants were instructed to record regular days, not, e.g., holidays/sick days where food intake was atypical. The food records were analyzed using the continuously updated Micro-Nutrica PC Program (Research and Development Unit of the Social Insurance Institution, Turku, Finland). The (P + M)/S (ratio of the intake of mono- and polyunsaturated to saturated fatty acids expressed as percentage of energy intake) and fiber intake (g/MJ) were assessed by combining dietary data collected from age 2 to 18 years. Participants were grouped into tertiles according to (P + M)/S and fiber intake in two steps. First, within four separate age intervals (2–6 years, 7–11 years, 12–16 years, and 17–18 years) the participant was defined as having a high (P + M)/S or high fiber intake in that interval if at least 50% of the age point measurements were in the highest tertile and none in the lowest tertile. Second, the participants with constantly high (P + M)/S or fiber intake were defined as having at least in 50% of the intervals a high, and never low, (P + M)/S or fiber intake. Participants with constantly low (P + M)/S or fiber intake were determined likewise.

The primary prevention of smoking was introduced in the intervention protocol at age 8 years. Since then smoking status was assessed by a questionnaire during each study visit [23]. Participants, who at the age of 20 years reported ever smoking regularly and had smoked at least 50 cigarettes during their life, were considered as smokers in the present study.

During the study visits, a physically active lifestyle was encouraged, but it was never a structured part of the intervention. Physical activity was assessed with a self-administered questionnaire when the participants were 13, 15, 17 and 19 years old. Leisure time physical activity was calculated as metabolic equivalent hours/week by multiplying the mean frequency, duration, and intensity of the reported physical activity. The mean value of metabolic equivalent measured between ages 13 and 19 years was used as an indicator of physical activity of the participant.

### 2.3. Physical Examination

Repeated physical examinations comprised measurements of weight, height, and BP. Weight was measured to the nearest 0.1 kg using an S10 electronic scale (Soehnle, Murrhardt, Germany) and height to the nearest 0.1 cm using a Harpender stadiometer (Holtain, Crymych, UK). BMI was calculated (weight per height squared; kg/m^2^). Childhood overweight/underweight was determined according to international criteria [30]. In adulthood, participants with BMI > 25 kg/m^2^ were determined as overweight/obese while BMI < 18.5 kg/m^2^ determined underweight. BP was measured once at 7 years of age, four times from 8 to 9 years of age, and twice at each visit thereafter using an oscillometric noninvasive BP monitor (Criticon Dinamap 1846 SX until 2001, thereafter Criticon Dinamap Compact T) and the mean value of the measurements was used to indicate BP. Paternal age at the time of the participant’s birth was queried.

### 2.4. Laboratory Measurements

Venous blood samples were collected after an overnight fast for determination of serum insulin, glucose, triglycerides, and total and high-density lipoprotein cholesterol (HDL-C). Low-density lipoprotein cholesterol (LDL-C) concentration was calculated using the Friedewald equation [31]. The samples were handled immediately and stored frozen until analyzes. All serum samples were analyzed at the laboratory of the National Public Health Institute in Turku, Finland. Serum total cholesterol concentration was analyzed with a fully enzymatic cholesterol oxidase p-aminophenazone method (Merck, Darmstadt, Germany) with an AU510 automatic analyzer (Olympus, Hamburg, Germany) or, after January 2001, with an AU400 analyzer [32]. Serum HDL-C concentration was measured after precipitation of apoB-containing lipoprotein particles by dextran sulfate 500,000 [33]. Samples used for the insulin and glucose concentration analyzes were centrifuged immediately. Details of the insulin and glucose analyzes have been reported previously [24].

### 2.5. Telomere Length Measurement

The primary outcome variables of the present study are the yearly TL attrition rate and the yearly change in the short telomere proportion. TL was measured twice during the intervention period. For the TL measurements, DNA was extracted from whole blood samples collected at a mean age of 7.5 years for the first measurement and 19.8 years for the second measurement by ethanol precipitation. Mean leukocyte TL and the short telomere proportion (<5 kB) were measured by Southern blot analysis of terminal restriction fragments using the Telo TAGGG TL assay kits (Roche Molecular Biochemicals, Basel, Switzerland) as previously described [34]. The mean TL attrition rate and the change in the short telomere proportion were calculated by subtracting the value of the first TL measurement from the value of the second TL measurement, and dividing the difference by the number of years between the TL measurements.

Samples for TL measures at both time points were available for *n* = 232 participants (intervention group: *n* = 108, control group: *n* = 124). Samples for successful assessment of the short telomere proportion at both time points were available for *n* = 213 participants (intervention group: *n* = 99, control group: *n* = 114). The time interval between the TL measurements ranged from 2 to 13 years (mean = 12.3 years), but was ≥10 years for 96.1%, and 13 years for 69.4% of the participants. The possible differences between the participants with TL measurements and those who remained in the study at age 7, but for whom TL measurements were not available (*n* = 482) were analysed. No differences were found in the main characteristics including sex, BMI, waist circumference, systolic BP and serum insulin concentration. A strong correlation was found between the first and the second measurement for both mean TL and the short telomere proportion (TL: r = 0.81, *p* < 0.0001; short telomere proportion: r = 0.80, *p* < 0.0001).

### 2.6. Genetic Risk Score Calculation

Genotyping was performed using the Illumina Cardio Metabochip custom array [35]. Imputation of missing genotypes was conducted using SHAPEIT v1 and IMPUTE2 software with the 1000 genomes as a reference panel [36,37,38]. We used the results of a recent large genome-wide association analysis to construct a weighted genetic risk score (wGRS) comprising seven genetic variants associated with leukocyte TL [6]. The score was calculated by summing the weighted number of genotyped alleles and imputed dosages of alleles associated with longer TL for each participant.

### 2.7. Statistical Analyses

The group and sex differences in the outcome variables were analyzed using Student’s *t*-test or linear regression adjusted for appropriate covariates. The associations between TL and continuous variables were analyzed using linear regression. The effect of exact age at both measurements was analyzed using sex-adjusted linear regression model. Mean values of dietary (P + M)/S and fiber intake from 2 to 18 years were summed, and for serum total and LDL cholesterol, ApoB and insulin concentrations, and systolic BP the mean values were calculated from measures between ages 7 and 20 years, and used for stepwise adjustments in modeling the intervention effect on TL. Additionally, the analyses were adjusted for baseline telomere length. A two-sided *p*-value < 0.05 was considered statistically significant. Statistical analyzes were performed using R version 3.4.3.

## 3. Results

### 3.1. Characteristics of the Study Population, Telomere Length Attrition Rate and Yearly Change in the Short Telomere Proportion

**Figure 1:**
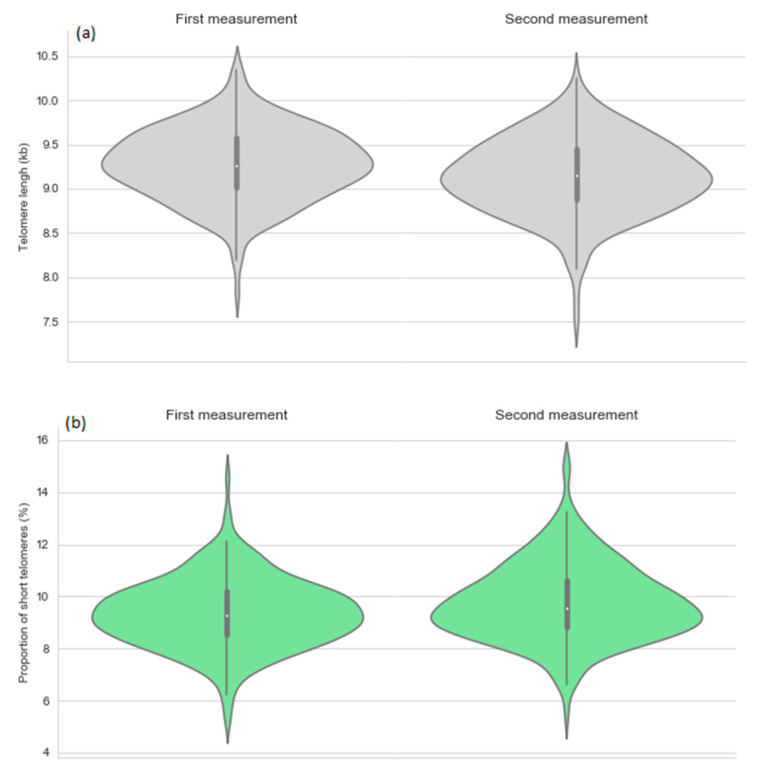
Telomere length (**a**) and proportion of short telomeres (**b**) by the first and second measurement point.

The characteristics of the intervention and control groups at the first and second TL measurement point are presented in the Table 1. Figure 1 depicts the TLs and proportions of short telomeres by the measurement point.

At both measurements the number of participants was N = 230–232 (intervention group N = 102–108; control group N = 118–124) for all other variables except fasting plasma glucose (N = 118; intervention group N = 57, control group N = 61) and insulin (N = 113; intervention group N = 52, control group N = 61) at the first measurement.

**Figure 2:**
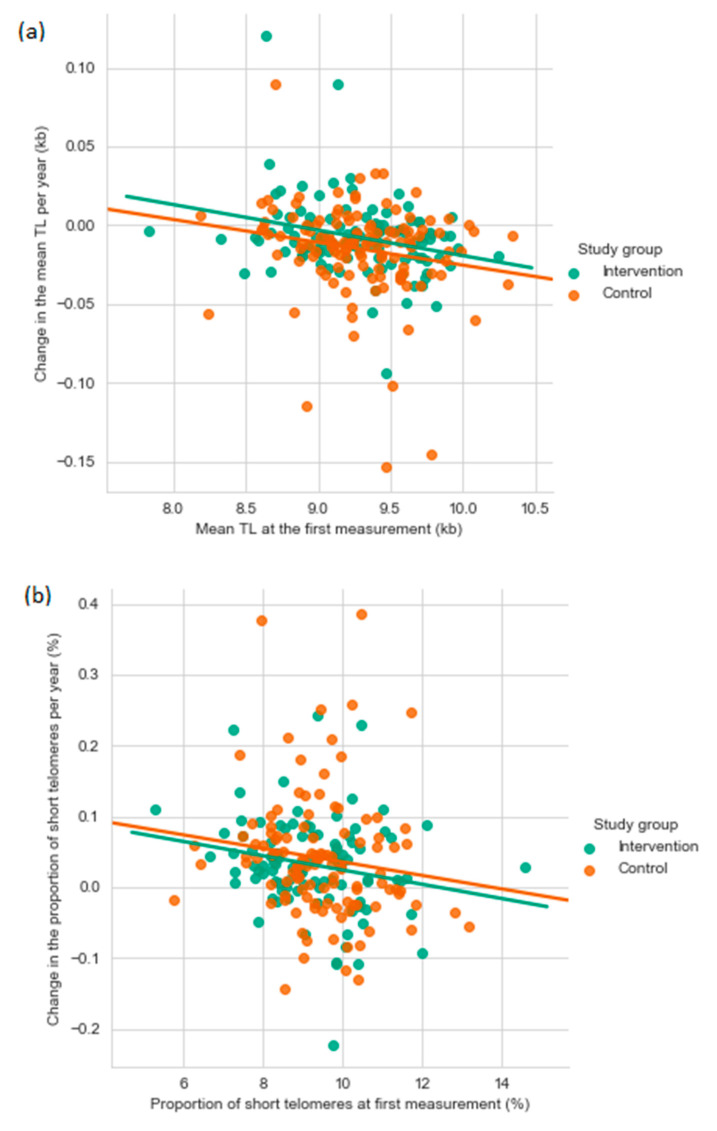
The yearly change in mean telomere length (**a**) and the percentage of short telomeres (**b**) as a function of the first mean TL or percentage of short telomere measurement. kb, kilobase; TL, telomere length.

**Figure 3:**
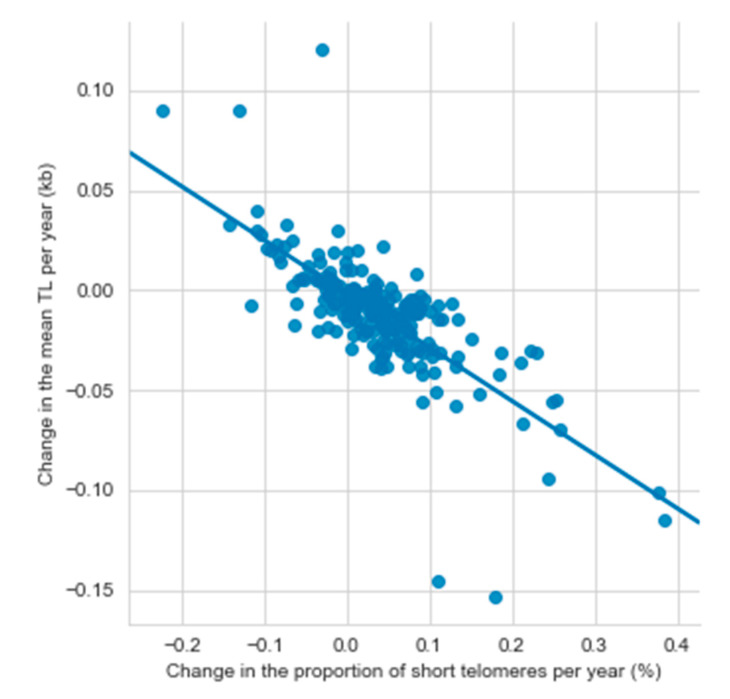
The relationship between changes in mean telomere length and the percentage of short telomeres per year (N = 232). kb, kilobase; TL, telomere length.

Among all participants, the mean TL attrition rate was 11.5 bp/year (SD 27.9), and it ranged from a decrease of 153 bp/year to an increase of 120 bp/year. An increase in TL was found in 23.7% of the participants. The mean increase in the short telomere proportion was 0.037%/year (SD 0.08) ranging from a decrease of 0.22%/year to an increase of 0.39%/year. The mean TL attrition rate and the yearly change in the short telomere proportion correlated negatively both with the TL at first measurement (TL attrition rate: r = −0.222, *p* = 0.0006; short telomere proportion: r = −0.167, *p* = 0.014) (Figure 2). A negative correlation between the mean TL attrition rate and the mean yearly change in the short telomere proportion (r = −0.749, *p* < 0.0001) indicated, that the short telomere proportion increased the most in the individuals with the fastest TL shortening (Figure 3).

### 3.2. Established Telomere Length Determinants

The associations of the previously established TL determinants (i.e., sex, age, paternal age and wGRS [2,5,6,7,8,39]) for TL and short telomere proportion were studied for the first and second TL measurement. Similar mean TL was observed among the girls and boys at the first (girls: mean = 9.30 kb, SD = 0.42 vs. boys: mean = 9.27 kb, SD = 0.38 kb, *p* = 0.467) and the second measurement (girls: mean = 9.17 kb, SD = 0.45 vs. boys: mean = 9.11 kb, SD = 0.40, *p* = 0.889). There were also no sex differences for the short telomere proportion at the first (girls: 9.29%, SD = 1.30 vs. boys: 9.43%, SD = 1.31, *p* = 0.365) and the second measurement (girls: 9.70%, SD = 1.44 vs. boys: 9.84%, SD = 1.46, *p* = 0.889). At the second measurement, an inverse association was observed between age and TL (β = −0.192, *p* = 0.002) as well as age and short telomere proportion (β = 1.009, *p* < 0.0001), while a direct association was found between paternal age and TL (β = 0.015, SE = 0.005, *p* = 0.003). There was no association between wGRS and TL or short telomere proportion in either measurement point.

### 3.3. Effect of STRIP Intervention on the Yearly TL Attrition Rate and the Yearly Change in the Short Telomere Proportion

The mean TL or the proportion of short telomeres was similar in the intervention and the control groups at the first or the second measurement in analyses adjusted for sex and age (Table 2; Model 1). When the analyses were additionally adjusted for factors related to the intervention targets (Model 2), the participants in the intervention group had a lower short telomere proportion compared to the controls at the second measurement.

**Figure 4:**
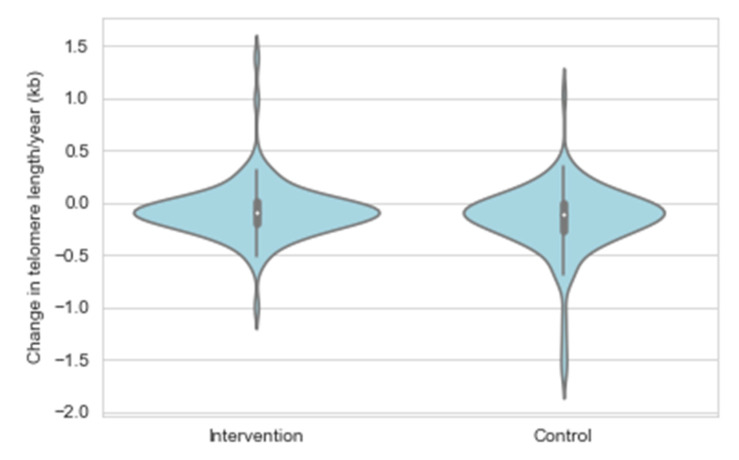
Change in telomere length expressed as a percentage of telomere length at the first measurement by the STRIP study group.

Age and sex adjusted linear regression analyses showed a significant intervention effect for the mean TL attrition rate (β = 0.007, SE = 0.004, *p* = 0.040) (Figure 4, Table 3, Model 1). In order to gain insights into the potential mechanistic pathways explaining the observed intervention effect, the age and sex adjusted model (Model 1) was further adjusted for variables related to the main intervention targets (Model 2: dietary fat and fibre intake, Model 3: serum total/LDL cholesterol and ApoB concentration, Model 4: insulin concentration, Model 5: systolic BP, and Model 6: physical activity and smoking) (Table 3). After that, all covariates were entered simultaneously into the same statistical model (Model 7). The intervention effect remained essentially unchanged in all separate models adjusted for potential confounders (β estimates varying between 0.006 and 0.011, *p* < 0.05 for the intervention effect in Models 2–6), but became stronger in the final model adjusting for all covariates (β = 0.013, SE = 0.005, *p* = 0.009). Intervention effect was not observed for the yearly change in the short telomere proportion.

## 4. Discussion

The key finding of this study is that the yearly TL attrition rate among the STRIP intervention participants was slower compared to the controls. The observed intervention effect was, however, not explained by the main features of the STRIP intervention such as dietary fat quality or intake of fiber, concentrations of serum lipids or insulin, or systolic BP. In contrast to the yearly TL attrition rate, the intervention did not have an effect on the change in the proportion of short telomeres, which may be explained by differences in biological mechanisms related to these two outcomes or by the small number of participants with short telomeres.

Only a few other longitudinal studies have addressed the association between diet and change in TL, and they have mostly been conducted in adults. Collectively, the data from these studies have been inconclusive. In a small group of overweight and obese children/adolescents, an energy-restricted diet was accompanied by a significant increase in TL [40]. In contrast, in middle-aged Finns with impaired glucose tolerance, a lifestyle intervention focusing on weight reduction and a reduction in total and saturated fat as well as an increase in fiber intake did not affect the 4.5 year change in TL during the active intervention or the post-intervention follow-up [41]. Another longitudinal observational study in young adults found an inverse association between baseline energy intake, and both baseline and follow-up TL, but not with TL shortening [42]. In 55 to 80-year-olds, adherence to a Mediterranean diet at baseline has been associated with longer telomeres in women, but no intervention effect was observed in the trial [21]. Recently, a longitudinal study among elderly Finns reported that diets such as the Baltic Sea diet and the Mediterranean diet had little impact on TL or telomere attrition [43]. In line with longitudinal studies, cross-sectional observations on diet and TL have also been inconsistent [15,16,17,18,19,20,21]. Notably, some of the studies support the importance of overall lifestyle patterns and a generally healthy lifestyle [9] while others concentrate on specific dietary factors, and report, e.g., that fiber or polyunsaturated fat intake has a favorable [15], and consumption of processed meat [16] or saturated fat [18] has an adverse association with TL. A recent review concluded that a healthy diet characterized by a high intake of dietary fiber and unsaturated fat likely exerts a protective role on telomere health whereas high consumption of sugar and saturated lipids accelerates telomere attrition [44].

As most studies addressing TL and TL attrition have been done in adults, the early life determinants of TL are still poorly understood. A direct association of dietary antioxidant capacity, and an inverse association of white bread consumption with TL has been found in children and adolescents aged 6–18 years [19]. Masi et al. (2012) found no association between leukocyte TL and traditional cardiovascular risk factors in adolescents, but reported an association between inflammatory markers, CRP and fibrinogen, and TL [45]. Other childhood risk factors associated with shorter TL include obesity [46], early-life stress and adverse childhood events [47], and physical inactivity [2].

Data from adults have shown that the TL attrition rate is inversely correlated with TL assessed at baseline [5,41]. Our results indicate that the same phenomenon can be observed already during the transition between childhood and young adulthood. The here observed 11.5 bp mean decrease in leukocyte TL per year is somewhat smaller than previously reported in young adults and elderly individuals [4,5]. However, it has been observed that the rate of telomere attrition vary markedly at different ages with high attrition rate during early childhood followed by a plateau from age four years until young adulthood [48]. It has also been suggested that telomere maintenance may be mainly genetically determined and modified by non-genetic factors throughout life-course [1]. Telomere attrition may, e.g., reflect the cumulative exposure to inflammation and oxidative stress, which are associated with incomplete replication of chromosomal ends through accelerated cell proliferation and DNA breaks that cause accelerated TL attrition [14]. When telomeres become critically short, cells enter replicative senescence [1]. Consequently, it has been proposed that, rather than the mean TL, abundance of short telomeres is the critical factor linked to telomere dysfunction and cellular survival [49]. For instance, in patients with type 1 diabetes progression of diabetic nephropathy has been more strongly associated with percentage of short telomeres than mean TL [35]. However, most human studies have focused on measuring the mean TL, and previous data on the effects of short telomeres especially in young healthy individuals are not available.

A potential limitation of the current study is the selection bias that may weaken the effects of the intervention. Even if our previous attrition analyses [23,27] have shown no differences in the main outcome variables between the participants and those who have dropped out, it is still possible that the families remaining in the study may be generally more health-conscious, or their lifestyle choices may have been modified by the regular study visits, even if they belonged to the control group. In addition, the STRIP trial was not originally designed to detect intervention effects on telomere biology, and the study may have had reduced statistical power as the sample size available for TL measurements at two time points was relatively small. We also acknowledge as a limitation that the samples were not available from baseline, at the beginning of the intervention, and they were not from exactly the same time points. Ideally, future studies would thus assess TL prior to beginning of the intervention to monitor the effect on the progression of TL. Future studies could also benefit from the availability of determinants of reactive oxygen species or autophagy to study the underlying factors for telomere modulation. Furthermore, we measured TL in leukocytes, similar to most studies, because they are easily accessible. The TL measured in leukocytes is, however, highly correlated with TL measured in other tissues within an individual [3] and reflect the senescence status of circulating cells of the immune system [1]. Evident strengths of the study are the longitudinal study design beginning from childhood, the ability to assess change in TL and proportion of short telomeres, and the unique, 20-year dietary intervention. Additionally, all participants were of similar ethnicity (white Caucasians), diminishing the effect of genetic predisposition to the observed results.

## 5. Conclusions

We found a small but statistically significant effect of the dietary and lifestyle intervention on the yearly TL attrition rate, but were unable to identify the specific intervention component underlying the observed effect. The finding that the STRIP intervention is reflected on attrition of telomere length in addition to the multifaceted favorable effects on cardiometabolic health at young age further adds to the clinical relevance of having a heart-healthy diet and lifestyle beginning from childhood.

## Figures and Tables

**Table 1:** Characteristics of the Special Turku Coronary Risk Factor Intervention Project (STRIP) intervention and control group participants with two measurements of leukocyte telomere length.

	First Measurement		Second Measurement	
	Intervention	Control	Intervention	Control
Age, years	7.50 ± 2.00	7.48 ± 2.00	19.8 ± 0.46	19.8 ± 0.44
BMI (kg/m^2^)	16.3 ± 2.00	16.3 ± 3.21	23.0 ± 3.50	23.0 ± 4.34
Overweight/obese (%)	16.7	14.5	18.5	21.8
Underweight (%)	6.5	15.3	4.6	8.1
Waist circumference (cm)	56.1 ± 5.45	56.4 ± 7.86	78.3 ± 9.00	77.7 ± 10.6
Systolic blood pressure (mmHg)	102 ± 9.56	101 ± 10.6	123 ± 12.0	120 ± 14.4
Diastolic blood pressure (mmHg)	59.0 ± 6.38	58.5 ± 6.55	66.8 ± 7.46	65.7 ± 8.16
Total cholesterol (mmol/L)	4.40 ± 0.65	4.56 ± 0.67	4.30 ± 0.76	4.36 ± 0.78
HDL-C (mmol/L)	1.28 ± 0.23	1.33 ± 0.25	1.31 ± 0.33	1.34 ± 0.33
LDL-C (mmol/L)	2.79 ± 0.53	2.90 ± 0.60	2.49 ± 0.58	2.54 ± 0.67
Triglycerides (mmol/L	0.69 ± 0.30	0.68 ± 0.26	1.11 ± 0.53	1.05 ± 0.52
Fasting plasma glucose (mmol/L)	4.47 ± 0.52	4.48 ± 0.46	4.96 ± 1.10	4.85 ± 0.63
Fasting serum insulin (mU/L)	4.61 ± 1.29	5.22 ± 3.60	11.4 ± 37.6	8.38 ± 9.73

Values are expressed as mean ± SD, unless otherwise indicated. BMI, body mass index; HDL-C, high-density lipoprotein cholesterol; LDL-C, low-density lipoprotein cholesterol.

**Table 2:** Mean leukocyte telomere length and short telomere proportion by the measurement point, and the STRIP intervention and control group.

		First Measurement			Second Measurement		
		Intervention	Control	*p*-Value	Intervention	Control	*p*-Value
Mean telomere length (mean, SD)	Model 1	9.27 (0.41)	9.29 (0.39)	0.81	9.16 (0.42)	9.12 (0.44)	0.42
	Model 2	9.30 (0.38)	9.29 (0.37)	0.49	9.19 (0.42)	9.11 (0.44)	0.080
Short telomere proportion (%)	Model 1	9.25 (1.38)	9.44 (1.27)	0.27	9.66 (1.44)	9.91 (1.46)	0.21
	Model 2	9.17 (1.36)	9.37 (1.19)	0.064	9.54 (1.29)	9.89 (1.49)	0.019

Model 1: adjusted for sex and age at measurement. Model 2: adjusted for sex, age, (P + M)/S-ratio, fiber intake, total cholesterol, apoB, LDL cholesterol, insulin, systolic blood pressure, physical activity, and smoking.

**Table 3:** Multivariable models of change in leukocyte telomere length (TL) and proportion of short telomeres by selected variables/targets of the STRIP intervention.

		Mean TL			Short Telomere Proportion		
		β Estimate (SE)	*p*-Value	N (Intervention/ Control)	β Estimate (SE)	*p*-Value	N (Intervention/ Control)
Model 1	Study group, age and sex	0.007 (0.004)	0.040	108/124	−0.011 (0.011)	0.310	99/114
Model 2	Model 1 + (P + M)/S-ratio + fiber intake	0.011 (0.005)	0.023	73/97	−0.022 (0.015)	0.146	67/88
Model 3	Model 1 + total cholesterol + apoB + LDL cholesterol	0.006 (0.003)	0.042	108/124	−0.011 (0.011)	0.332	99/114
Model 4	Model 1 + insulin	0.007 (0.040)	0.039	108/124	−0.011 (0.011)	0.308	99/114
Model 5	Model 1 + systolic blood pressure	0.007 (0.004)	0.044	108/124	−0.011 (0.011)	0.327	99/114
Model 6	Model 1 + physical activity + smoking	0.008 (0.004)	0.027	105/119	−0.014 (0.011)	0.221	96/111
Model 7	All models combined	0.013 (0.005)	0.009	71/93	−0.029 (0.015)	0.065	65/85

All analyses for mean TL and the proportion of short telomeres were additionally adjusted for the respective baseline value. For dietary variables ((P + M)/S; poly- and monounsaturated to saturated fatty acids ratio, and fiber) an average value for variables assessed between ages 2 and 18 years was calculated and used in the analyses. For total cholesterol, LDL cholesterol, apoB, and insulin concentrations as well as for systolic blood pressure the average value from all measurements from age 7 to 20 years was calculated and used in the model. For physical activity, the average value of metabolic equivalent h/wk at ages 13, 15, 17 and 19 years. Participants who at age 20 years reported ever smoking regularly and had smoked altogether at least 50 cigarettes were considered as smokers. SE, Standard Error.

## Data Availability

The rights to the data belong to the STRIP research group. Selected variables and their descriptions without personal identification codes are distributed to investigators and collaborators working on specific projects. Data sharing outside the STRIP group requires a data-sharing agreement. Investigators can submit an expression of interest to the STRIP Steering Committee.
